# Type I fatty acid synthase trapped in the octanoyl‐bound state

**DOI:** 10.1002/pro.3797

**Published:** 2020-01-11

**Authors:** Alexander Rittner, Karthik S. Paithankar, Aaron Himmler, Martin Grininger

**Affiliations:** ^1^ Institute of Organic Chemistry and Chemical Biology, Buchmann Institute for Molecular Life Sciences Goethe University Frankfurt Frankfurt am Main Germany

**Keywords:** cooperativity, fatty acid synthesis, inhibition, multienzyme, substrate‐bound state, transacylation

## Abstract

De novo fatty acid biosynthesis in humans is accomplished by a multidomain protein, the Type I fatty acid synthase (FAS). Although ubiquitously expressed in all tissues, fatty acid synthesis is not essential in normal healthy cells due to sufficient supply with fatty acids by the diet. However, FAS is overexpressed in cancer cells and correlates with tumor malignancy, which makes FAS an attractive selective therapeutic target in tumorigenesis. Herein, we present a crystal structure of the condensing part of murine FAS, highly homologous to human FAS, with octanoyl moieties covalently bound to the transferase (MAT—malonyl‐/acetyltransferase) and the condensation (KS—β‐ketoacyl synthase) domain. The MAT domain binds the octanoyl moiety in a novel (unique) conformation, which reflects the pronounced conformational dynamics of the substrate‐binding site responsible for the MAT substrate promiscuity. In contrast, the KS binding pocket just subtly adapts to the octanoyl moiety upon substrate binding. Besides the rigid domain structure, we found a positive cooperative effect in the substrate binding of the KS domain by a comprehensive enzyme kinetic study. These structural and mechanistic findings contribute significantly to our understanding of the mode of action of FAS and may guide future rational inhibitor designs.

**Abbreviations**
ACP: acyl carrier protein
DH: dehydratase
ER: enoylreductase
FAS: fatty acid synthase
KR: ketoreductase
KS: β‐ketoacyl synthase
LD: linker domain
MAT: malonyl‐/acetyltransferase
TE: thioesterase

## 1 INTRODUCTION

Fatty acids are essential molecules in most living cells, serving as key compounds of cell membranes, as energy supply in the metabolism, as secondary messengers in signaling pathways or as covalent modifications to recruit proteins to membranes. They can either be obtained directly from the diet or are synthesized de novo by fatty acid synthases (FASs) from simple building blocks in repeating cyclic reactions. Although the chemistry of fatty acid synthesis is largely conserved across all kingdoms of life, the structural organization of the participating enzymes differs dramatically. FAS complexes occurring in plants, bacteria, and in mitochondria, known as the Type II, perform biosynthesis by a series of monofunctional separate enzymes.^1^, ^2^, ^3^ In contrast, the CMN group bacteria (*Corynebacterium*, *Mycobacterium*, and *Nocardia*), fungi and higher eukaryotes utilize Type I FASs that integrate all enzymatic functions into large macromolecular assemblies.^4^, ^5^, ^6^, ^7^ Fungal and CMN‐bacterial FASs form up to 2.7‐MDa α_6_β_6_‐heterododecameric barrel‐like structures.^8^, ^9^, ^10^, ^11^, ^12^ The animal FASs, including human FAS, emerge from a separate evolutionary development and consist of two polypeptide chains assembling into a 540‐kDa intertwined “X‐shaped” homodimer.^13^

**Figure 1:**
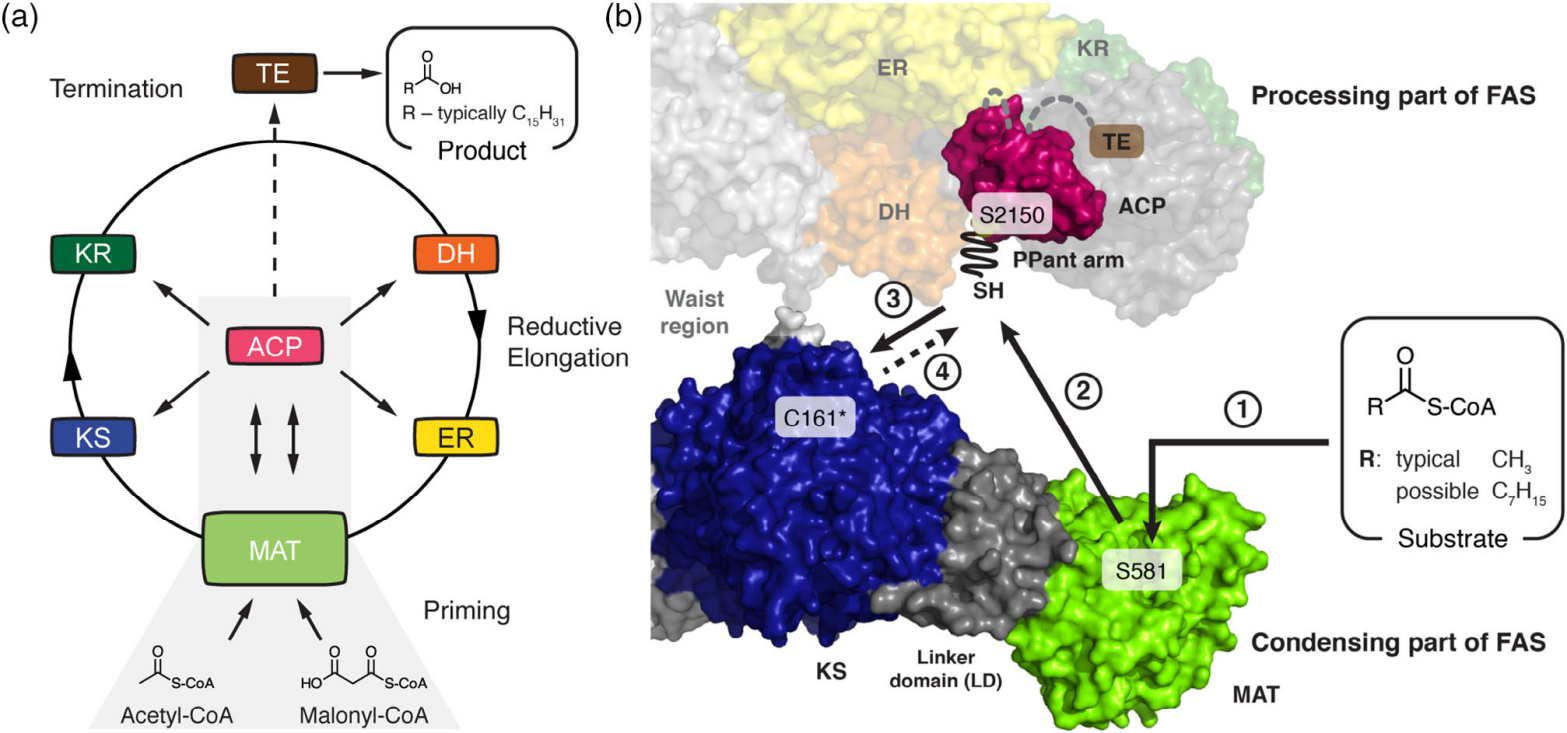
(a) Priming of animal fatty acid synthesis. (b) In a first step, the substrate is selected by the MAT domain and transferred to the ACP domain (Step 2) from where it is passed on to the KS domain (Step 3). Important active site residues are highlighted and C161 is marked with an asterisk. Crystal structure of porcine FAS (PDB code http://firstglance.jmol.org/fg.htm?mol=2vz9) and NMR structure of rat ACP (PDB code http://firstglance.jmol.org/fg.htm?mol=2png) are depicted in surface representation.^13^, ^14^ Domains of one protomer of FAS homodimer are colored. ACP, acyl carrier protein; DH, dehydratase; ER, enoylreductase; FAS, fatty acid synthase; KR, ketoreductase; KS, ketosynthase; MAT, malonyl‐/acetyltransferase; PPant arm, expand fully in the figure; TE, thioesterase

In animals, including human beings, fatty acid biosynthesis commences with the transfer of an acetyl moiety from acetyl‐coenzyme A (CoA) to the terminal thiol of the phosphopantetheine arm of the acyl carrier protein (ACP) domain catalyzed by the malonyl‐/acetyltransferase (MAT) domain (Figure 1a).^15^ After being passed on to the active site cysteine of the β‐ketoacyl synthase (KS) domain, a malonyl moiety is loaded on the free ACP domain in a second MAT‐mediated transfer reaction. Upon delivery of the malonyl moiety, the KS domain catalyzes a decarboxylative Claisen condensation reaction in which the KS‐bound acetyl and the ACP‐bound malonyl moieties combine to an ACP‐bound β‐ketoacyl intermediate. Subsequently, the β‐keto group is sequentially modified by three processing domains, the ketoreductase (KR), the dehydratase (DH), and the enoylreductase (ER) using NADPH as a reducing agent. Typically, fatty acid synthesis runs seven cycles to deliver a fully reduced ACP‐bound acyl chain of 16 carbon atoms, which is eventually released as palmitic acid by hydrolysis via the thioesterase (TE) domain (Figure 1a).

The key domains in fatty acid synthesis are MAT, KS, and ACP, responsible for the selection of substrates, C─C bond formation and substrate shuttling, respectively (Figure 1b). The MAT domain of murine Type I FAS shows broad substrate specificity and facilitates the synthesis of methyl‐branched, odd numbered, and functionalized fatty acids by alternative substrate selection.^16^, ^17^, ^18^, ^19^ On the contrary, the KS domain possesses a strict specificity for saturated acyl moieties with a low acceptance of β‐keto groups to guarantee biosynthesis of saturated fatty acids in vivo.^20^ The ACP domain is loaded by the MAT and ACP does not impose substrate specificity in this step. However, the interaction of ACP with other domains is necessary for the progress of synthesis and the specificity of this interaction can affect the product formation.^21^, ^22^, ^23^

Although FASs are ubiquitously expressed in all tissues, de novo biosynthesis of fatty acids occurs at low levels as the demand is usually met by the diet.^24^, ^25^ Despite adequate nutritional lipid supply, the FAS gene is overexpressed under pathological conditions, being associated with diseases like diabetes, obesity, and cancer, and upregulation of FAS correlates with tumor malignancy.^26^, ^27^, ^28^, ^29^, ^30^, ^31^ FAS has emerged as a very promising therapeutic target in tumorigenesis, because pharmacological FAS inhibitors induce tumor cell death by apoptosis, whereas normal cells are resistant.^32^ To date, several inhibitors, like cerulenin, GSK2194069, and orlistat, have been identified or developed that target the KS, KR, and TE domain, respectively.^32^, ^33^, ^34^ Remarkably, the compound TVB‐2640 recently entered Phase 2 clinical trials showing promising results in combinatorial anticancer therapies.^35^, ^36^

Herein, we report the crystal structure of the murine KS‐MAT didomain at 2.7 Å resolution with octanoyl moieties covalently bound in the KS and the MAT active sites. By comparison with structures of domains in apo‐form as well as with the malonyl‐bound MAT domain, we analyze structure–function relationships and correlate the conformational variability of the individual domains with their substrate specificities. Furthermore, by applying a continuous fluorometric assay, we reveal detailed mechanistic insight into the cooperative behavior of the KS‐mediated transacylation reaction. The results of this study provide new insights into the key processes of substrate loading and condensation in fatty acid synthesis and foster the development and optimization of inhibitors with potential antineoplastic properties.

## 2 RESULTS

### 2.1 Crystal structure of the KS‐MAT didomain with bound octanoyl moieties

**Figure 2:**
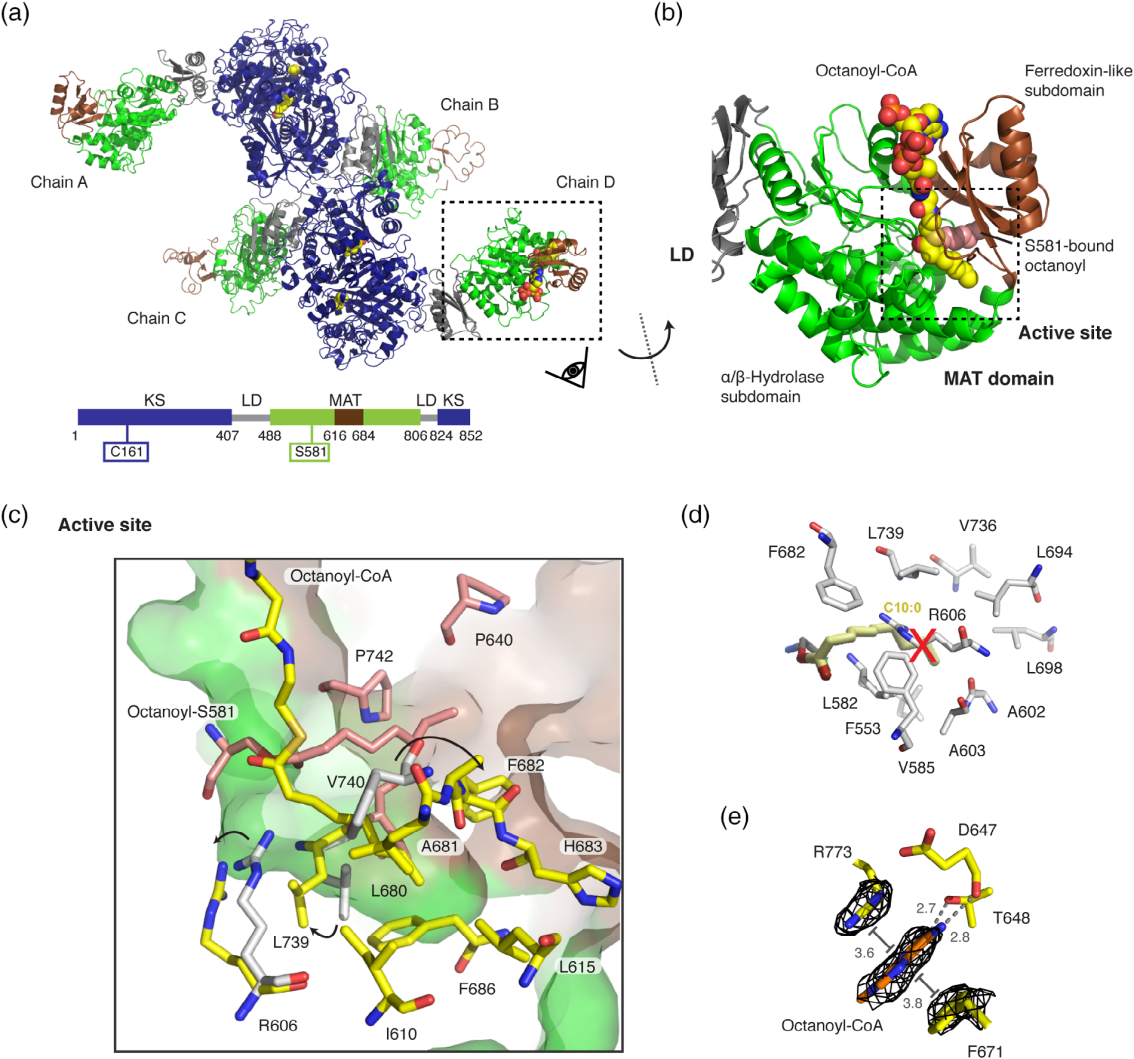
Octanoyl‐loaded MAT domain. (a) Polypeptide chains in the unit cell with bound octanoyl moieties in yellow in sphere representation. Domains and folds are colored as depicted in the attached cartoon. (b) Zoom into the MAT domain in chain D. The active site is embedded in a cleft between the α/β‐hydrolase (green) and the ferredoxin‐like (brown) subdomains. The active site serine (S581) was found in an octanoyl‐bound state with an additional octanoyl‐CoA molecule non‐covalently attached to the active site tunnel (see Figure S2 for FEM^38^ and Polder maps^39^). (c) Zoom into the MAT active site. Residues interacting with the serine‐bound octanoyl chain and the octanoyl‐CoA are colored in red and yellow, respectively. Movements of select residues upon binding of an octanoyl moiety in comparison with the human MAT structure (gray) are indicated by arrows. (d) Orientation of a decanoyl chain as reported by Bunkoczi et al.^40^ Atomic coordinated originate from decanoyl chain computationally modeled into the human MAT variant R606A (PDB code: http://firstglance.jmol.org/fg.htm?mol=2jfd). Postulated interacting residues of the human MAT domain are shown in gray. (e) Binding site of the nucleobase of the CoA moiety at the MAT surface. The adenine is coordinated via hydrogen bonding with residues D647 and T648 and via π‐stacking and π‐cation interactions with residues F671 and R773 were identified. FEM, feature‐enhanced map; MAT, malonyl‐/acetyltransferase

In order to gain structural insights into the molecular basis for the substrate ambiguity of the MAT domain and the strict substrate specificity of the KS domain of murine Type I FAS, we aimed at trapping both KS and MAT domains in the octanoyl‐bound enzyme state. Following an established protocol,^16^ the purified murine KS‐MAT didomain, sharing 87% sequence identity to the condensing part of human FAS,^37^ was crystallized and crystals were soaked with octanoyl‐CoA (Figure S1). X‐ray diffraction data were collected to a resolution of 2.7 Å, and the resulting structural model refined to *R*/*R*_free_ of 0.18/0.23 (Table 1). The asymmetric unit contains four polypeptide chains (A–D) arranged as two biological dimers interacting via the cleft between the KS and the linker domain (LD) (Figure 2a). In all four chains, the KS domain is modified with an octanoyl moiety yielding the octanoyl‐enzyme covalent complex. Furthermore, in one polypeptide chain (chain D), an octanoyl group is covalently bound to S581 of the MAT domain and an additional octanoyl‐CoA is non‐covalently trapped in the MAT binding tunnel (Figure 2b). This finding confirms previous data showing that octanoyl‐CoA can prime murine Type I fatty acid synthesis and that the MAT domain can catalyze the transfer of octanoyl moieties.^16^, ^17^

**Table 1:** Data collection and refinement statistics

	Wildtype soaked with octanoyl‐CoA
*Data collection*	
Space group	C222_1_
Cell dimensions	
*a*, *b*, i (Å)	147.4, 354.0, 218.5
*α*, *β*, *γ* (°)	90, 90, 90
Resolution (Å)	50–2.7 (2.75–2.7)
No. of reflections	2,195,612 (110,844)
*R*_meas_	0.16 (2.4)
*I*/*σI*	13.9 (1.4)
CC_1/2_	0.99 (0.62)
Completeness (%)	98.2 (98.8)
Redundancy	14.3 (14.5)
*Refinement*	
*R*_work_/*R*_free_ (%)	18.4 (23.2)
No. of unique reflections	145,548
*Average B‐factors* (*Å* ^2^)	
Protein	78.9
Wilson *B* factor	58.4
*RMSD from ideality*	
Bond length (Å)	0.007
Bond angles (°)	1.5
*Ramachandran statistics*	
Favored regions (%)	93.43
Allowed regions (%)	5.05
Outliers (%)	1.52

*Note*: Highest resolution shell is shown in parentheses.

### 2.2 General description of the octanoyl‐bound MAT domain

The MAT domain engages fatty acid synthesis in selecting the CoA‐ester substrates for product assembly. It is located at the edge of the condensing part of animal FAS and inserted into the KS fold via the LD. The exposed position and the utilization of only 8.4% of the solvent‐accessible area for domain–domain interactions reflect a high structural independence from the FAS fold. The substrate‐binding pocket is formed by a cleft between the α/β‐hydrolase and the ferredoxin‐like subdomains and extends to the active site located in the center of the domain. The function of the MAT domain is to shuttle acyl moieties via the active serine between CoA‐ester substrates and the ACP domain following a ping–pong bi–bi mechanism. The catalytic key residues S581 and H683 form a catalytic dyad with S581 acting as nucleophile and H683 serving in acid–base catalysis.^41^ The nucleophilicity of S581 is enhanced by a helix dipole‐moment due to its positioning within a strand‐turn‐helix motif termed the nucleophilic elbow.^42^ A key residue for the bifunctional role of MAT is R606 located in helix 7, which interacts with the carboxylic group of extender substrates. The absence of the guanidinium group leads to altered substrate specificity.^16^, ^43^

Upon soaking protein crystals with octanoyl‐CoA, we found that S581 in chain D is covalently modified with an octanoyl moiety and, furthermore, a molecule octanoyl‐CoA is non‐covalently attached to the substrate binding pocket (Figure 2b). The placement of both ligands was based on the feature‐enhanced map (FEM) and later validated by a Polder map (Figure S2).^38^, ^39^ The octanoyl chain of octanoyl‐S581 is located in a tunnel between the two subdomains created between residues P640, F682, V740, and P742 and extends to the protein's surface (Figure 2c). The octanoyl group of octanoyl‐CoA points toward helix 10 of the α/β‐hydrolase fold in a substrate binding pocket between the subdomains created by residues I610, L615, L680, A681, F682, H683, F686, and L739. Rangan and Smith reported that the R606A variant from rat FAS showed increased turnover rates for the transfer of octanoyl moieties.^43^ Based on this, Bunkoczi and co‐workers^40^ placed a decanoyl chain in the human R606A‐mutated MAT binding site by a simulated docking experiment and concluded that space for longer acyl chains is created in the mutant due to the absence of the side chain of residue R606. In our structure, the positions of the octanoyl chains are slightly different to that of the computationally docked decanoyl chain. Upon binding octanoyl‐CoA, the side chains of residues R606 and L739 rotate to form an extended binding cavity that can accommodate the larger substrate (see Figure S3 for a stereo view). This feature is likely responsible for the high transacylation rate of murine MAT for octanoyl moieties and may not be present in rat MAT according to docking data.^40^ Again deviating from rat FAS data,^43^ the murine MAT loses the capability for efficient transacylation of the octanoyl moiety upon mutation of R606 to alanine (Table 2).

**Table 2:** Kinetic analysis of the transacylation reaction with octanoyl‐CoA at a fixed acceptor concentration of 60 μM ACP (*n* = 4)

Substrate	*K*_m_^app^ (μM)	*k*_cat_^app^ (s^−1^)	*k*_cat_/*K*_m_ (M^−1^ s^−1^)	Hydrolysis rate 10^−3^ (s^−1^)
Wildtype^a^	0.7 ± 0.3	4.1 ± 0.3	5.6 ± 2.2 × 10^6^	6.1 ± 1.0
R606A variant	4.9 ± 0.7	0.037 ± 0.008	7.6 ± 2.0 × 10^3^	2.3 ± 3.4

Abbreviation: ACP, acyl carrier protein.

a Previously published.^16^

Intriguingly, in the MAT‐octanoyl‐CoA complex, the nucleobase of octanoyl‐CoA is bound at a specific position between the two subdomains with the adenine stacked between side chains of F671 and R773. Besides the well‐known π‐stacking between aromatic rings, also π‐cation interactions between arginines and aromatic rings are known.^44^ Additionally, two hydrogen bonds are formed between amines of the purine ring to both the side chain hydroxyl group of T648 and the backbone carbonyl group of D647.

### 2.3 Implications on MAT subdomain dynamics from crystal structures

**Figure 3:**
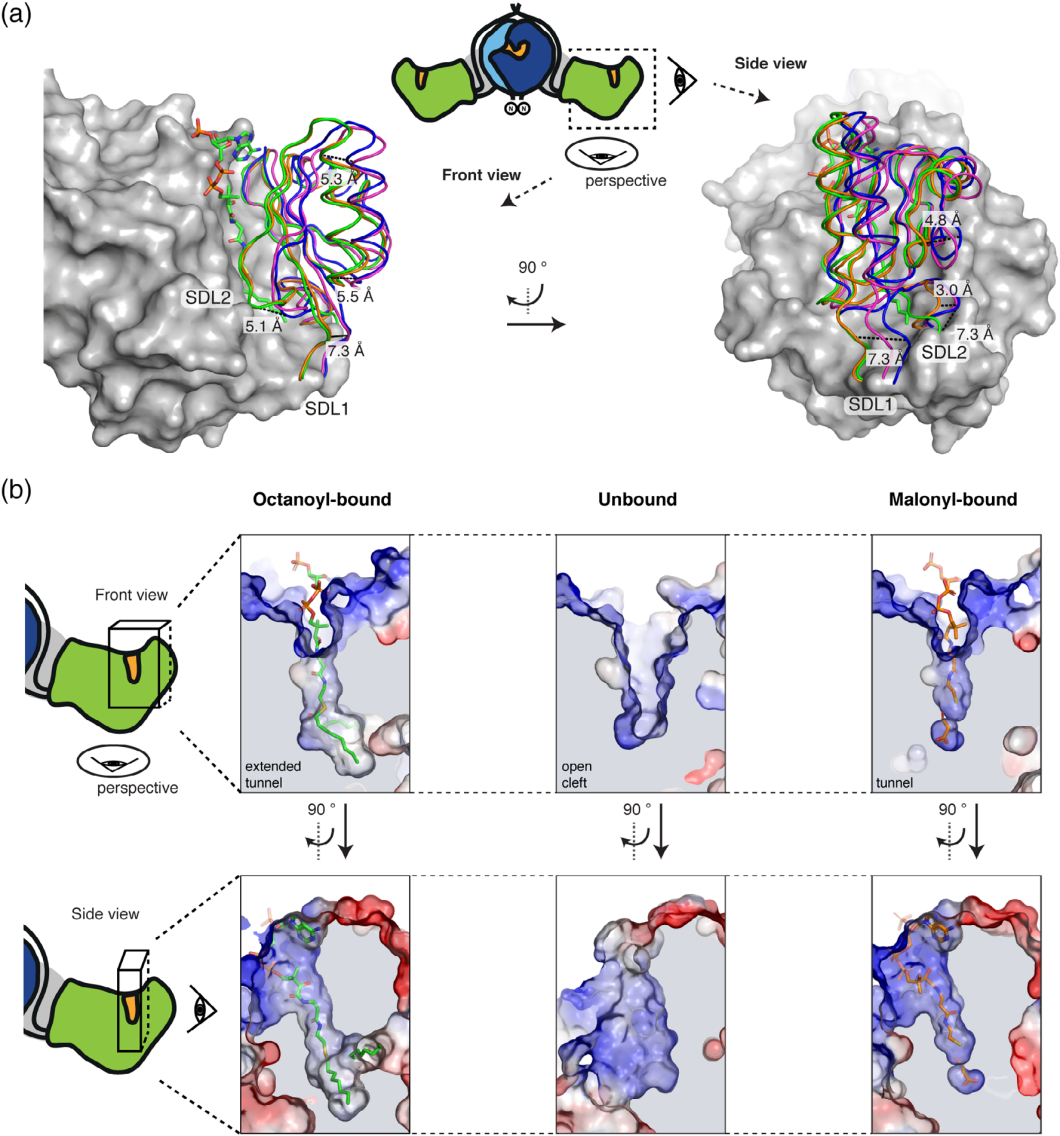
Conformational variability of the MAT active site. (a) α/β‐Hydrolase fold centered superposition (BB of residues 488–613 and 685–806) of four MAT domains in different acyl‐bound states. chain A (blue) and chain D (green) from the octanoyl‐CoA soaked crystal (PDB code: http://firstglance.jmol.org/fg.htm?mol=6rop), malonyl‐bound (orange) (PDB code: http://firstglance.jmol.org/fg.htm?mol=5my0; chain D) and apo human MAT (purple) (PDB code: http://firstglance.jmol.org/fg.htm?mol=3hhd; chain A) were used. The α/β‐hydrolase subdomain is shown in surface depiction and the ferredoxin‐like fold in cartoon loops. Selected distances between corresponding residues indicate the mobility of the subdomains with a relative movement of the ferredoxin‐like fold of up to 7.3 Å. (b) Different active site and entry tunnel shapes upon substrate binding. Surface depictions of active sites of chain D (left panel) and A (middle) from the octanoyl‐bound structure and chain D (right panel) from the malonyl‐bound structure are shown in two perspectives. Surfaces are shown in surface electrostatic representation calculated with PyMOL and shown in default coloring with positive potential depicted in blue and negative potential in red. Views as in (a). BB, backbone atoms; MAT, malonyl‐/acetyltransferase

Variations in the relative positioning of the ferredoxin‐like subdomain were reported in previous crystal structures, but a correlation of subdomain mobility to the substrate ambiguity of the domain could not yet be drawn. In chain D of the MAT‐octanoyl‐CoA complex, the MAT domain was found in a unique conformational state. Keeping the α/β‐hydrolase part of the domain (backbone atoms [BB] of D488–D611 and D685–D806) as a reference, a superposition was performed with the apo‐structure in chain A, the malonyl‐bound structure (PDB code: http://firstglance.jmol.org/fg.htm?mol=5my0; chain D) and the human KS‐MAT (PDB code: http://firstglance.jmol.org/fg.htm?mol=3hhd; chain A). The α/β‐hydrolase domain superimposes very well in all the four models with RMSDs (BB) to the MAT‐octanoyl‐CoA complex of 0.6 (chain A), 0.8 (Malonyl‐bound chain D), and 0.6 (human chain D) Å, respectively (Figure 3a). Largest differences are found in the relative positioning of the ferredoxin‐like subdomain with local shifts of up to 7.3 Å between corresponding residues. As also the ferredoxin‐like subdomains (BB of 618–674) of all four models themselves superimpose well with RMSDs (BB) between 0.4 and 0.8 Å, these results clearly illustrate that the ferredoxin‐like subdomain describes a rigid‐body movement.

When the static X‐ray structural information is subjected to a TLS (translation, libration, and screw) refinement, the derived anisotropic displacement parameters imply a rotational movement describing the opening and closing of the active site cleft to allow binding of diverse substrates (Figure S4).^45^ In order to determine residues contributing most to the positional variability of the ferredoxin‐like and the α/β‐hydrolase subdomains, we plotted main‐chain torsion angles *φ* and *ψ* (Ramachandran plot) for the MAT domains of the various structural models. The plot identifies residues A613 and H614 as well as H683 and S684 as undergoing significant changes in main‐chain torsion angles (Figure S5). Both sites are the hinges of two subdomain linkers, termed SDL1 (612–617) and SDL2 (675–684), allowing movements of SDL1 and SDL2 of about 7.3 and 5.1 Å, respectively (Figure S6). The positional and conformational variability of the subdomain linkers allows changes in the relative orientation of the subdomains and in the geometry of the active site cleft for the accommodation of chemically and structurally diverse CoA‐esters (Figure 3b).^16^

In addition to the overall dynamics of the MAT fold, the residue R606, responsible for holding the carboxyl group of extender substrates, shows high positional variability in the MAT structural models. The high degree of rotational freedom of the side chain originates likely from the specific property of animal MAT in featuring a phenylalanine at a position (F553, murine MAT numbering), which is otherwise occupied by a conserved glutamine. As shown previously, F553 significantly diminishes the coordination of the R606 side chain by hydrogen bonding.^16^ In the octanoyl‐bound structure, we could identify a third rotameric state of R606, in addition to the ones found in apo‐ and malonyl‐bound state (Figure S7), which demonstrates that the adaptation of the domain to different substrates is closely connected to the rotational variability of this residue.

### 2.4 Structure of the KS domain in an acylated state

**Figure 4:**
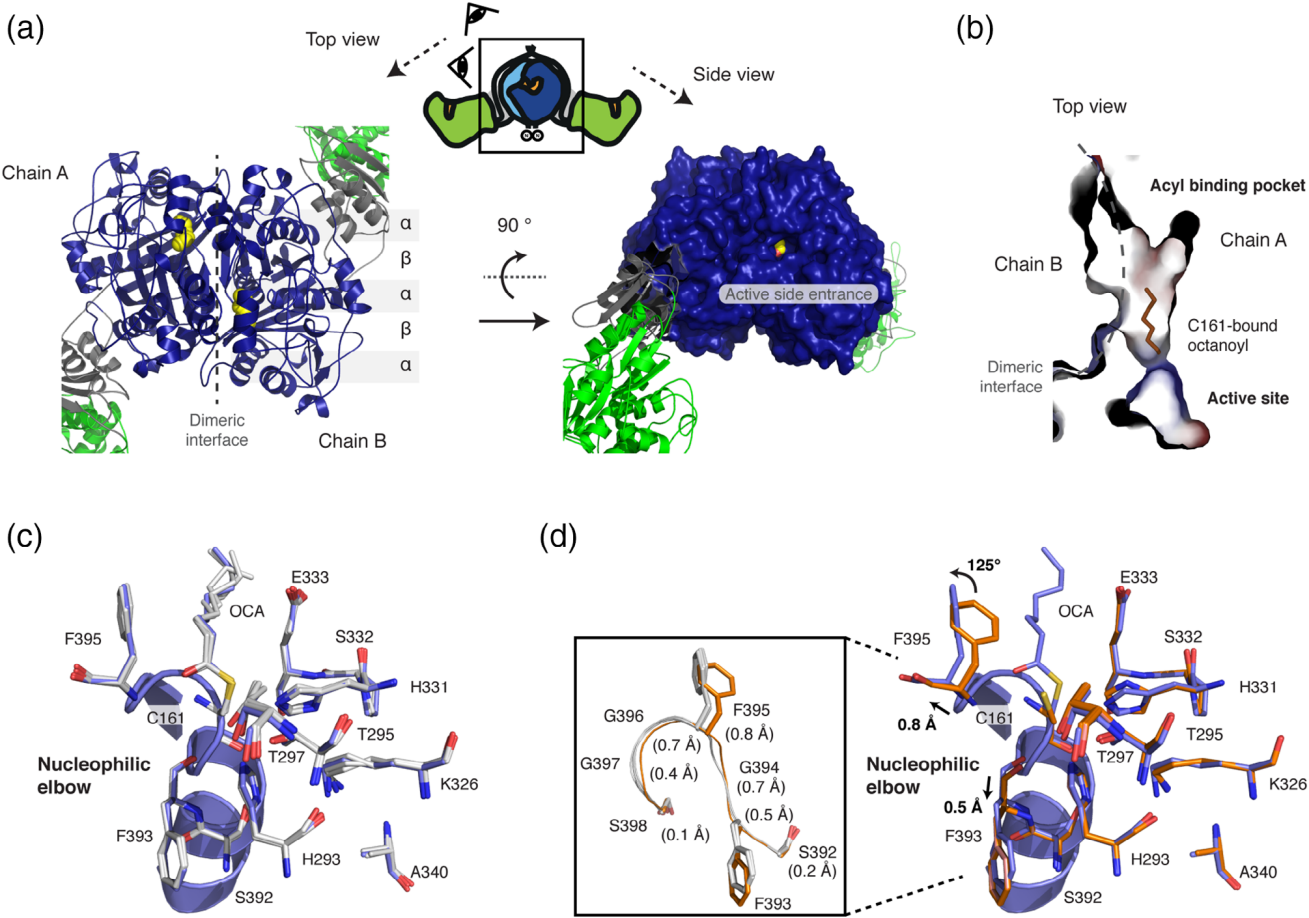
Octanoyl‐loaded KS domain. (a) Top view on the dimeric KS domain in cartoon depiction showing the topology of the α/β/α/β/α sandwich motif (left panel). A surface depiction of the KS domain in side view highlights the active site entrance. Color codes as in Figure 2a are used with the bound octanoyl chain shown in yellow in sphere representation (right panel). (b) Active site and acyl binding cavity of the KS domain. In addition to the substrate binding cavity at the dimer interface, a small side chamber is visible in the monomer. The binding cavity is shown with surfaces colored in electrostatic potential (colored as in Figure 3). (c) Active site of KS showing important residues for catalysis, reported for homologous KAS I (FabB).^46^ Three chains (b–d) with bound octanoyl moieties were aligned to chain A (blue) by a KS based superposition (BB of residues 1–407 and 824–852). All residues adopt essentially the same conformation with some variability in the terminal carbon atoms of the octanoyl chain. (d) A similar KS based superposition was performed with the four apo‐KS domains (orange; PDB code: http://firstglance.jmol.org/fg.htm?mol=5my0) and the octanoyl‐bound chain A (blue). Upon octanoyl binding, the individual residues of the stretch 393–397 are shifted by 0.4–0.8 Å (highlighted in the inlet). Furthermore, the side chain of F395 is rotated by approximately 125°. BB, backbone atoms; KS, ketosynthase

The KS domain forms dimers in Type I FAS systems, and contributes the largest area (about 2,580 Å^2^; see Table S1 for more information) to the overall dimerization interface of animal FAS. The KS domain belongs to the thiolase‐superfamily and exhibits the characteristic topology of alternating layers of α‐helices and β‐sheets (called α/β/α/β/α sandwich motif) (Figure 4a). A small vestibule in lateral orientation to the twofold axis of the condensing part forms the entry to the active site, which is comprised of the active cysteine (C161) as well as two histidine (H293, H331) residues, termed the catalytic triad. The substrate binding tunnel further extends toward the dimer interface, where it merges with the tunnel of the protomer at the twofold axis (Figure 4b).

Overall all the four polypeptide chains of the asymmetric unit align very well onto one another when a KS domain based superposition is performed (RMSDs [BB] of about 0.20–0.25 Å over the residue ranges 1–407 and 824–852; Figure 4c). All four active sites in the KS domains are modified with octanoyl moieties at residue C161. The position and conformation of all active site residues are essentially identical. Only the bound octanoyl chain shows positional variability in the terminal carbon atoms due to an unconstrained rotational freedom of the single bonds. Taking also into account the overall low B‐factors observed in the KS part of the crystal structure, this data indicates a relatively low degree of flexibility within the KS domain.

As observed for S581 in the MAT domain, the active cysteine C161 is located in a nucleophilic elbow, where the positive dipole‐moment of the α‐helix decreases the p*K*_a_ value of the thiol group sufficiently to be readily deprotonated under physiological condition. Although widely accepted, this helix effect was relativized in a more recent computational study, where a zwitterionic state is considered as active with H293 accepting the proton from C161 via a bridging water molecule.^47^ The closer H331 on the other hand acts as a general acid in the second step of the catalysis. Here, nitrogen (ND1) accepts a hydrogen bond from backbone amide E333 (3.0–3.4 Å) and hence the protonated nitrogen (NE2) is in hydrogen bond distance (3.2–3.4 Å) to the sulfur of the thioester bond at residue C161.

Furthermore, the bound octanoyl chains allow localization of the oxyanion hole, which is created by backbone amides of residues C161 and F395. In all chains, the carbonyl's oxygen of the thioester is in hydrogen bond distance to the corresponding amides of F395 (2.9–3.1 Å) and slightly further apart from backbone amides of C161 (3.1–3.4 Å).

Next, we were interested in conformational changes induced by the loading of an octanoyl chain in comparison to the unbound state. Therefore, the four unbound murine KS domains of the unit cell (PDB code: http://firstglance.jmol.org/fg.htm?mol=5my0) were aligned to octanoyl‐bound chain A (serving as the representative chain) in a KS domain based superposition (residues 1–407 plus 824–852; Figure 4d). Again, overall RMSDs (BB) were small (0.2–0.3 Å), but the superposition revealed distinct differences in the positions and side chain conformations of some residues. Most prominently, the stretch of residues FGFGG (residues 393–397) is slightly shifted and reorganized upon binding of the octanoyl moiety. This results in the displacement of F395 by 0.8 Å (between corresponding carbon_α_ atoms) plus the rotation of the side chain by approximately 125°. F395 in the rotamer position of the unbound state clashes with the octanoyl chain in the bound state implying that the rearrangement of F395 is necessary to accommodate substrates.

**Figure 5:**
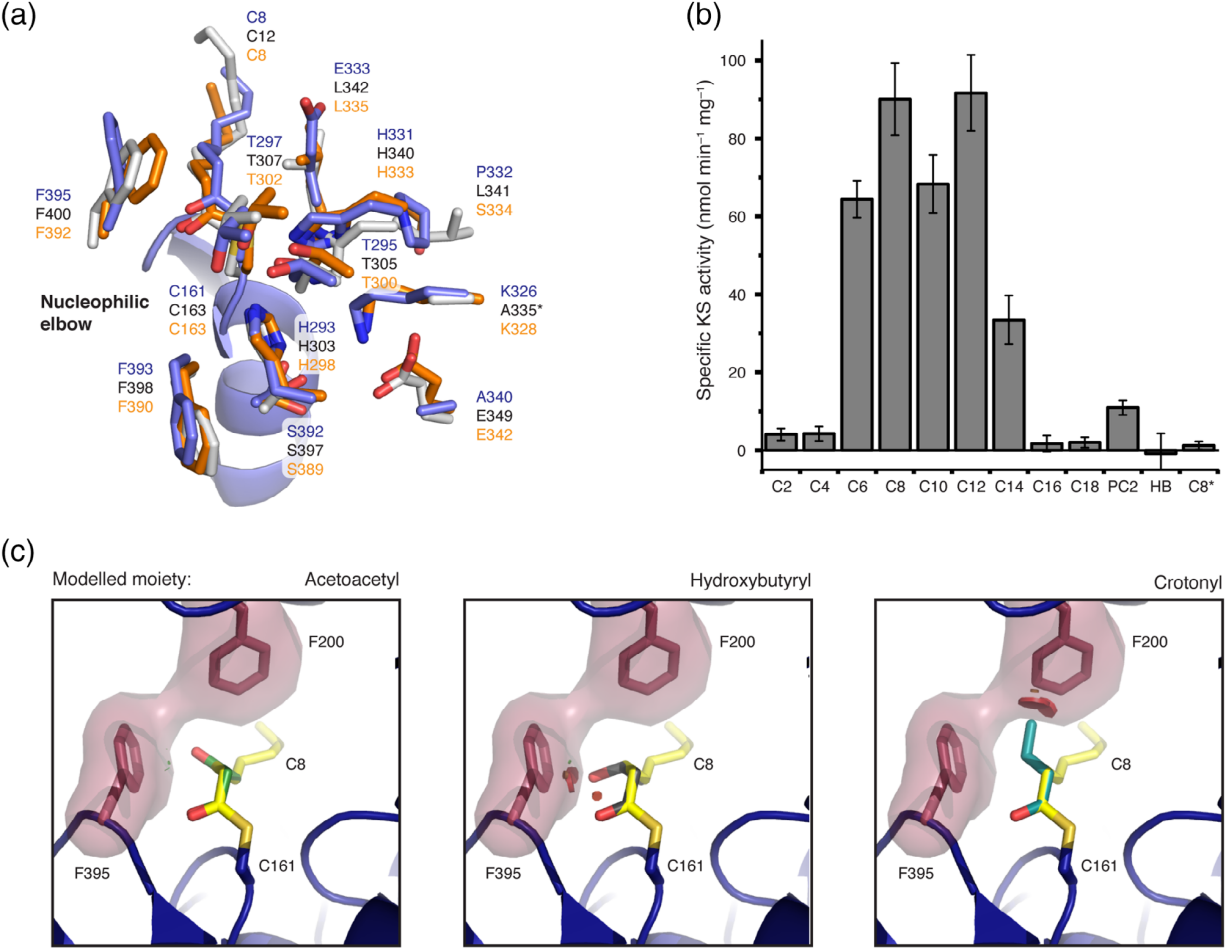
Chain‐length specificity of KS and comparison of the KS domain with FabB and FabF from *E*. *coli*. (a) Comparison of important active site residues of the murine Type I KS domain (chain A; blue) with FabB (Orange; PDB code: http://firstglance.jmol.org/fg.htm?mol=2bui) and FabF (gray; PDB code: http://firstglance.jmol.org/fg.htm?mol=2gfy) from *E*. *coli*.^48^, ^49^ All three proteins were solved in the acyl‐bound state and *E*. *coli* proteins were aligned to chain A (blue) by a KS based superposition (BB of residues 1–407). The asterisk indicates that a variant of FabF was crystalized possessing a K335A mutation. (b) Chain‐length specific KS‐mediated transacylation activity. The specific KS‐mediated activity was determined at fixed substrate (500 μM) and holo‐ACP (75 μM) concentrations using the αKGDH‐assay. The asterisk indicates usage of variant KS^C161G^MAT^S581A^ as negative control. Abbreviations refer to acyl‐CoA esters with different chain lengths and PC2 and HB refer to phenylacetyl‐CoA and hydroxybutyryl‐CoA, respectively. (c) Substrate selectivity of the KS domain by gating of the active site through the gatekeeper residue F395 and F200 (both red). The three moieties acetoacetyl (green), hydroxybutyryl (gray), and crotonyl (cyan), common intermediates of fatty acid synthesis, were modeled in place of the bound octanoyl moiety. Clashes are highlighted by red bumps. ACP, acyl carrier protein; BB, backbone atoms; KS, ketosynthase

FabB (KAS I) and FabF (KAS II), both elongating β‐ketoacyl‐ACP synthases of the bacterial Type II fatty acid synthesis, have been well‐characterized in their three‐dimensional structure in an octanoyl‐bound state and in a dodecanoyl‐bound state, respectively (see, e.g., References 46, 48, 49). KS domain based superpositions of FabB and FabF to chain A (BB residues 1–407) show that the active sites display an overall identical architecture implying a conserved catalytic mechanism for the Type I KS domain (Figure 5a). An exception is a glutamate residue, which is conserved in CHH class structures (E342 and E349 in FabB and FabF, respectively) and is thought to participate in catalysis by stabilizing a water or cation molecule.^46^, ^48^ This acidic residue is exchanged by an alanine in the Type I KS domain (A340) excluding an equivalent role in animal FAS.

### 2.5 Specificity of the KS domain for saturated acyl chains

The first step in the KS‐mediated Claisen condensation is the transacylation of an acyl‐moiety from acyl‐ACP to the active site cysteine (Figure 1). Considering the similarity of this step to the MAT‐mediated transacylation, we aimed at using the continuous fluorometric assay, originally established for transferase analysis,^16^, ^50^ to investigate the substrate specificity of the KS domain. In doing so, we have constructed KS‐MAT^S581A^ for specific KS read‐out and the double knockout mutant KS^C161G^‐MAT^S581A^ as a control. All didomain constructs proved to be stable, which was validated by size‐exclusion chromatography (SEC) profiles and by melting temperatures obtained in a thermal shift assay (Figure S8). We determined KS‐mediated turnover rates from various acyl‐CoA esters to a separated, standalone holo‐ACP at fixed substrates concentrations (Figure 5b). The experiment generally confirmed the results from Witkowski et al. of turnover rates increasing with acyl chain‐length until maximum rates are reached for octanoyl‐CoA and dodecanoyl‐CoA (C12‐CoA), and decreasing rates with chain‐length above C12‐CoA.^20^ The high value for C12‐CoA is not consistent with previous results for the rat homolog and seems to be a specific feature of the heterologously expressed murine KS domain. The specificity of the transfer of acyl moieties was confirmed with the KS^C161G^‐MAT^S581A^ double knockout mutant (Figure 5b).

Further, the substrate specificity of the KS domain was probed with two non‐cognate acyl‐CoA substrates. While the hydroxybutyryl‐CoA, mimicking the intermediate after an initial reduction by the KR‐domain, was not accepted as substrate, the non‐canonical compound phenylacetyl‐CoA was transferred with a reasonable rate. The latter result confirms our previous data that this substrate can also serve as a priming substrate for fatty acid synthesis.^17^

To draw a simple structure–function relationship for the substrate specificity of the KS domain, we modeled the three moieties acetoacetyl, (*R*)‐3‐hydroxybutyryl and crotonyl in place of the octanoyl moiety (Figure 5c). These are common intermediates of fatty acid synthesis and can either be accepted with low rates (acetoacetyl) or are not tolerated significantly (hydroxybutyryl and crotonyl). Structural data confirms the selectivity, as the keto group of the acetoacetyl moiety only slightly clashes with F395, whereas the hydroxyl group of hydroxybutyryl shows strong clashes with F395. A crotonyl moiety binds differently and shows strong clashes with F200.

### 2.6 The KS‐mediated transacylation shows kinetic cooperativity

To gain insight into the enzymatic properties of the KS domains, the absolute kinetic parameters for the KS‐mediated transacylation from acyl‐CoA esters to the ACP domain (as a standalone protein) were determined by the assay described before. The KS‐mediated transacylation reaction follows a ping–pong bi–bi mechanism with a covalently bound acyl‐enzyme intermediate and can be described with the general Equation (1) that is based on standard Michaelis–Menten kinetics.^51^ In order to determine absolute kinetic parameters, we have used this equation to globally fit two series of response curves with octanoyl‐CoA and myristoyl‐CoA at five or six different fixed ACP concentrations (see Section 4). The global fit could only moderately describe the dependence of the apparent turnover rates in respect of the individual ACP concentrations and disclosed systematic deviations in the response curves at low and high substrate concentrations (see Figure S9).

**Figure 6:**
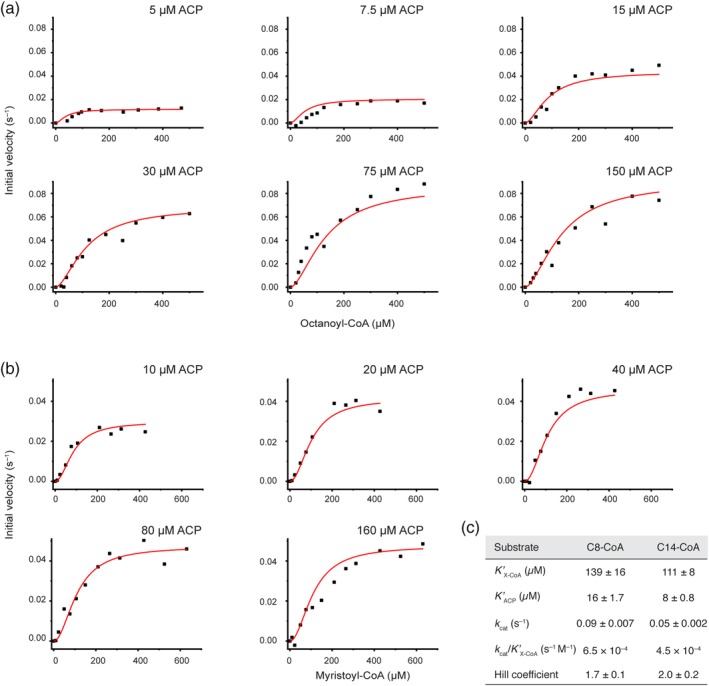
Comprehensive analysis of the KS‐mediated transfer of octanoyl and myristoyl moieties. (a) Initial velocities plotted against octanoyl‐CoA (C8‐CoA) concentrations at six fixed ACP concentrations. (b) Initial velocities plotted against myristoyl‐CoA (C14‐CoA) concentrations at five fixed ACP concentrations. All data series were fit globally with the Hill equation due to the sigmoidal shape. (c) Absolute kinetic parameter derived from the respective global fits for octanoyl‐CoA (C8‐CoA) and myristoyl‐CoA (C14‐CoA), respectively. No parameters constraints were imposed during curve fitting. The constant *K*′ of the Hill equation is related to the Michaelis constant *K*_m_, but also contains terms related to the effect of substrate occupancy at one site on the substrate affinity of the other site. ACP, acyl carrier protein; KS, ketosynthase

For a better description of the sigmoidal shape of the individual plots, and considering the dimeric nature of the KS domain, we included a Hill coefficient for both substrate concentrations (CoA‐Ester and ACP) as described in Equation (2). This new fit function clearly delineates both data series without imposing any parameter constraints (see Figure 6). The absolute kinetic constants (*K′*) and turnover numbers (*k*_cat_) for octanoyl‐CoA and myristoyl‐CoA are 139 ± 16 μM (*K′*), 0.09 ± 0.007 s^−1^ (*k*_cat_), and 111 ± 8 μM, 0.05 ± 0.002 s^−1^, respectively. These values lead to specificity constants (*k*_cat_/*K′*) in the range of 10^2^–10^3^ s^−1^ M^−1^, which indicate rather inefficient priming of the KS domain by CoA‐esters. The *K′*_ACP_ of the standalone ACP was with 16 ± 1.7 μM higher for the transfer of octanoyl moieties than for myristoyl moieties (8 ± 0.8 μM). The calculated Hill coefficients were between 1.7 and 2 for both substrates indicative of a positive cooperativity of the KS domains of a dimeric unit during the transacylation reaction.

## 3 DISCUSSION

We recently determined kinetic parameters for the murine MAT‐mediated transfer of canonical and non‐canonical acyl‐CoA substrates illustrating the broad substrate tolerance of this domain.^16^ How can this property be explained, considering high structural conservation to highly specific acyltransferases, like for example, FabD of *E*. *coli*? The presented ensemble of MAT structures with non‐covalently bound acyl‐CoA and covalently bound acyl moieties shines light on this peculiarity. When considering the individual structures as snapshots of an overall conformational variability, data reveal significant dynamics within the MAT domain. Since soaking with octanoyl‐CoA trapped the MAT in a very unusual conformation, revealing significant alterations in the position of active site residues, the newly presented structure is particularly informative in this respect. The data show that substrate polyspecificity of MAT originates from the overall high relative positional dynamics of the α/β‐hydrolase and the ferredoxin‐like subdomain. A pronounced conformationally variability of the subdomain linkers SDL1 and SDL2, embedded in this large‐scale movement, is further relevant (Figure 3) for the accommodation of the chemically and structurally diverse substrates. Finally, residue R606 modulates substrate polyspecificity by either swinging out to liberate space for the acyl chain (e.g., octanoyl moieties), or by coordinating to the free carboxylic group of extender substrates (e.g., malonyl moieties; Figure S7). This structural interpretation is supported by enzyme kinetic data as the (wildtype) murine MAT domain shows higher substrate ambiguity than the R606A‐mutated MAT domain. In fact, the wildtype MAT domain is even able to accept octanoyl moieties with significantly higher efficiency than the R606A construct,^16^, ^40^ which could possibly be explained by a smaller number of populated conformational enzyme states (Table 2).^52^

Can the rather strict KS domain substrate specificity be observed in structural properties? Indeed, the KS domain shows minor structural changes upon binding of an octanoyl chain, resembling a key‐and‐lock type binding, possessing strict specificities, as also observed for Type II systems.^48^ The most prominent conformational change upon acylation with saturated acyl chains emerges from the stretch of residues 393–397, in particular residue F395, which is consequently slightly shifted in position and rotated in the side chain by approximately 125°. Its postulated role as a “gatekeeper” seems to be confirmed in animal FAS, as functional groups (keto and hydroxy) in the β‐position of a bound acyl chain would sterically clash with the phenyl ring (Figure 5c).^47^, ^53^ In the evolutionarily strongly related protein class of polyketide synthases (PKSs), which share a common KS domain fold, residues at the F395‐equivalent position vary, reflecting the key feature of PKSs of condensing β‐keto‐, β‐hydroxy‐, and α‐β‐unsaturated acyl substrates.^54^

Our setup using a continuous coupled enzyme assay offers a convenient way to investigate the specificity of the KS domain in‐depth. The kinetic characterization generally revealed maximum transacylation rates for CoA‐esters of medium chain lengths (C8–C12) and confirmed earlier results of Witkowski et al. (Figure 5b).^20^ The specific activities for the short acyl chains were lower than expected, which may be attributed to the usage of CoA‐esters as donors and hence substrate concentrations that were insufficient to fully saturate the enzyme. Titration of acyl‐CoA substrates at different fixed ACP concentrations for octanoyl‐ and myristoyl‐CoA resulted in sigmoidal individual initial velocity curves (Figure 6). Global fitting of the individual curves was possible when including Hill coefficients for both substrates and the obtained Hill coefficients of 1.7–2 indicate positive cooperativity. This data can generally be interpreted by an increase in the efficiency of transferring an acyl chain to the active site cysteine of the KS domain, when the other KS of the dimer is already occupied. Such cooperativity of the domains of a KS dimer was postulated for the Type II homologs^48^ and could be explained by a conformational interconnection of both active sites that are pointing toward each other and merging at the twofold axis.

**Figure 7:**
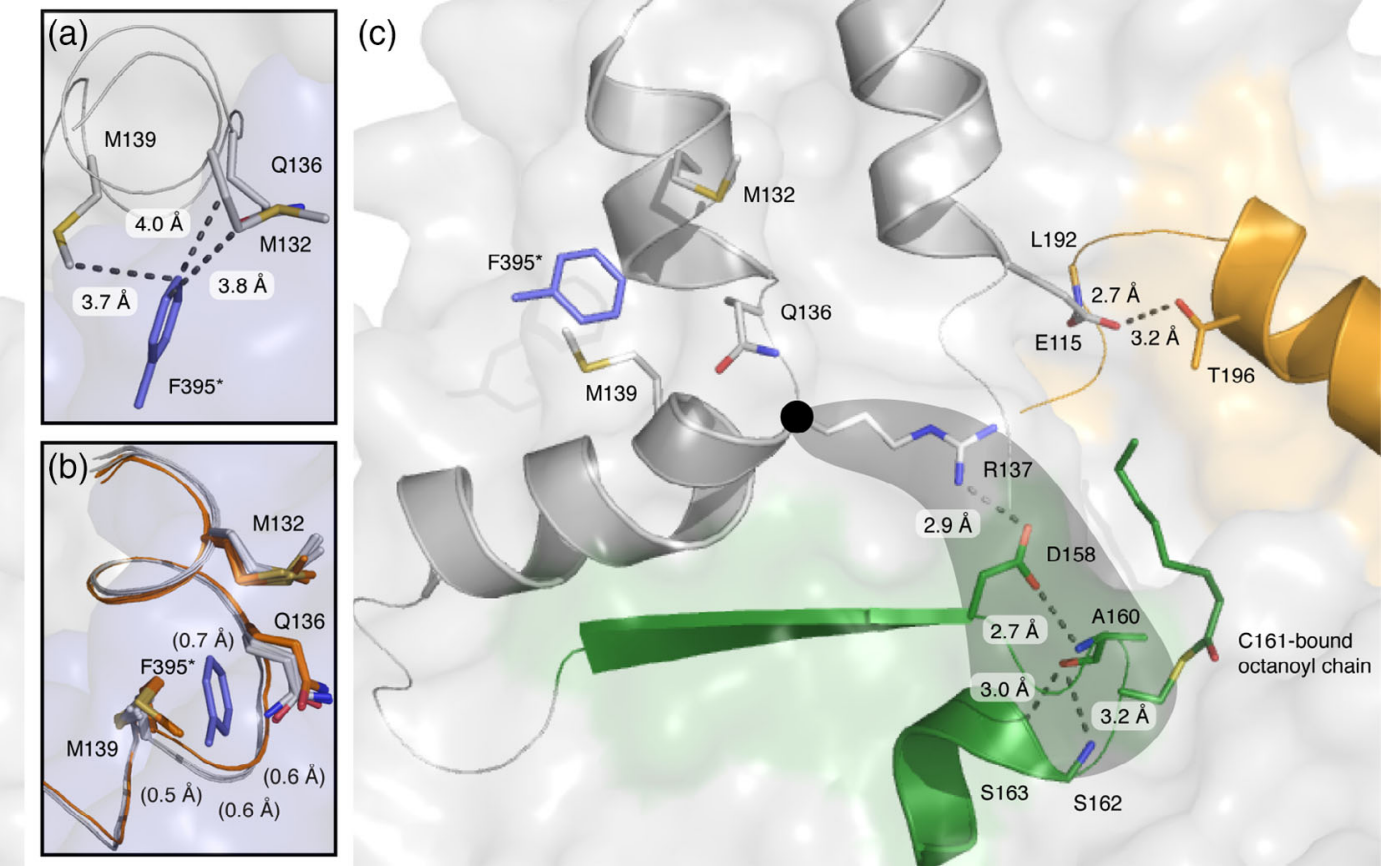
Structural interconnection of both KS active sites of a dimer. (a) Identification of M132, Q136, and M139 as residues interacting with F395 of the biological (KS–KS) dimer. Chains A and B are colored in gray and blue, respectively. (b) Side chain conformations adopted by M132, Q136, and M139 with a subtle backbone shift between unbound (orange) and octanoyl‐bound (grey) KS active sites. All four chains of both crystal structures (5my0 and 6rop) were aligned by a KS based superposition (BB of residues 1–407). F395 (blue) is depicted for clarity and the asterisks indicates the hypothetical position of the residue in the respective other protomer. (c) Hydrogen bonding between R137, D158, and the nucleophilic elbow (green) including the active site residue C161. BB, backbone atoms; KS, ketosynthase

Whereas a direct interaction of bound substrates can be ruled out as the origin for cooperativity, because the enzyme bound acyl intermediates are too far apart from each other (15.6 Å between terminal C‐atoms of octanoyl chains), a stretch of residues (residues 393–397) harboring the gatekeeping F395 and shifting upon acylation may be responsible for this phenomenon. The residues are residing at the dimeric interface and interact with a helix‐turn‐helix motif of the adjacent protomer (Figure 7a). In the acylated state, side chains of residues M132, Q136, and M139 (adjacent protomer) are slightly altered in their positions due to the rotation of F395, which furthermore leads to a slight shift of the turn (of the helix‐turn‐helix motif) by up to 0.7 Å (Figure 7b). Structural data reveals a putative coupling of this local rearrangement with active site residues via two hydrogen bonds; one between the side chains of R137 and D158 and the other between the carboxy group of D158 and the backbone amide of A160 (Figure 7c). All three residues are fully conserved in FASs. Based on this structural analysis, cooperativity could hence originate from a subtle reorganization of the active site residues in the neighboring protomer essentially induced by structural changes at the dimer interface occurring during acylation. Further experiments need to elaborate the molecular basis for the observed cooperative behavior of the KS domain as well as to analyze whether cooperativity observed for the transacylation step also extends to the Claisen condensation step.

The specific kinetic information about the MAT and KS domains allow us to vividly draw the murine FAS function *in vivo*. The direct loading of the KS domain with acyl‐CoA is not relevant *in vivo*, because the specificity constants of the KS‐mediated transfer of acyl moieties from acyl‐CoAs is more than three orders of magnitude lower than of the respective MAT‐mediated transfer.^16^ Accordingly, substrates are in general loaded at ACP by MAT‐mediated transacylation. ACP‐bound acetyl‐ and butyryl‐moieties are then transferred relatively slowly from the ACP domain to the KS domain and can in a competing pathway escape from FAS by MAT‐mediated offloading. Release of short acyl‐CoAs via the MAT domain depends on the *in vivo* ratio between malonyl‐CoA, acetyl‐CoA, and free CoA and hence the availability of an empty MAT's active site. With increasing malonyl‐CoA concentrations at higher energetic state of the cell, the offloading event of acetyl‐ and butyryl‐moieties gets more and more unlikely and further elongation becomes dominant.^55^ As shown here, once a chain‐length of six carbons and longer is reached, transacylation from the ACP domain to the KS domain becomes highly efficient until it sharply drops with a chain‐length of 16 carbons (Figure 5b). According to a key finding of this study, the efficiency of FAS in the elongation of the acyl chain is increased when both reaction chambers are used simultaneously, as acylation of one KS domain of the FAS dimer accelerates the acylation of the other (Figure 6). The substrate specificity of the KS domain is important for the specific production of palmitic acid and is assisted by the substrate specificity of the TE domain for long fatty acyl intermediates.^5^, ^56^, ^57^, ^58^ In summary, FAS produces short acetyl and butyryl‐CoA esters in a low energetic state of the cell and almost exclusively palmitic acid (C16) at high energetic states.

The presented structural and mechanistic study deepens our molecular understanding of the two initial catalytic domains in animal fatty acid synthesis. Such detailed information is particularly interesting as FAS has come into focus as a target for combinatorial anticancer therapy. Especially, the plasticity of the MAT domain shall be highlighted, which allows the accommodation of a broad range of chemically diverse compounds. This may aid future rational drug discovery campaigns and enlarge the pool of potentially screened lead structures.

## 4 METHODS AND MATERIALS

### 4.1 Reagents and constructs

All CoA‐esters, β‐NAD^+^, NADH, α‐ketoglutarate dehydrogenase (porcine heart; αKGDH), α‐ketoglutaric acid, thiamine pyrophosphate (TPP), and EDTA were purchased from Merck. BSA was from Serva. Restriction enzymes were bought from NEB biolabs. IPTG was from Carl Roth. Ni‐NTA affinity resin was from Qiagen and 5 ml Strep‐Tactin® columns were purchased from IBA technologies. Purity of CoA‐esters was confirmed by HPLC‐UV analysis before usage.

Point mutations were introduced by PCR based cloning. Fragments for pAR159 and pAR160 were generated by amplification of pAR69 (Addgene, #122849) and pAR70^16^ with the primer pair: AR301 (5′‐gtgcccaatgatgccgtcag‐3′) and AR310 (5′‐ggcatcattgggcacGccttgggagaggttgcctgtgg‐3′; see Table S2). PCR products were treated with Dpn1 (NEB), purified by gel electrophoresis and DNA was extracted with the Wizard® SV Gel and PCR Clean‐Up System (Promega). Purified DNA was transformed into *E*. *coli* Stellar™ Competent Cells, 5 ml LB cultures were grown and plasmids were isolated with the PureYield™ Plasmid Miniprep System (Promega). Sequences of all plasmids were confirmed with the “dye terminator” method.

### 4.2 Expression and purification of KS‐MAT variants

All plasmids were transformed into chemically competent *E*. *coli* BL21 Gold (DE3) cells. The transformants were grown overnight at 37°C in 20 ml LB (100 μg ml^−1^ ampicillin [amp] and 1% [w/v] glucose) medium. Precultures were used to inoculate 1 L TB medium (100 μg ml^−1^ amp). Cultures were grown at 37°C until they reached an optical density (OD_600_) of 0.5–0.6. After cooling at 4°C for 20 min, cultures were induced with 0.25 mM IPTG, and grown for additional 16 hr at 20°C and 180 rpm. Cells were harvested by centrifugation (4,000 rcf for 20 min). The cell pellets were resuspended in lysis buffer (50 mM potassium phosphate, 200 mM potassium chloride, 10% [v/v] glycerol, 1 mM EDTA, 30 mM imidazole [pH 7.0]) and lysed by French press. After centrifugation at 50,000 rcf for 30 min, the supernatant was mixed with 1 M MgCl_2_ to a final concentration of 2 mM. The cytosol was transferred to Ni‐NTA‐columns and washed with five column volumes (CV) wash buffer (lysis buffer without EDTA). Bound protein was eluted with 2.5 CV elution buffer (50 mM potassium phosphate, 200 mM potassium chloride, 10% [v/v] glycerol, 300 mM imidazole [pH 7.0]). The eluent was transferred to Strep‐Tactin‐columns, and washed with five CV strep‐wash buffer (50 mM potassium phosphate, 10% [v/v] glycerol, 1 mM EDTA [pH 7.0]). Proteins were eluted with 2.5 CV elution buffer (strep‐wash buffer containing 2.5 mM D‐desthiobiotin). After concentration to 10–20 mg ml^−1^, the proteins were frozen in liquid nitrogen and stored at −80°C. Samples were thawed at 37°C for 30 min and further polished by SEC using a Superdex 200 GL10/300 column equilibrated with the strep‐wash buffer. Proteins were concentrated again to 10–20 mg ml^−1^ and stored frozen in aliquots using liquid nitrogen.

### 4.3 Expression and purification of ACP

Acyl carrier protein for the activity assay was produced by co‐expression with the 4′‐phosphopantetheinyl transferase Sfp from the bicistronically organized vector pAR352.^16^ The plasmid was transformed into chemically competent *E*. *coli* BL21 Gold (DE3) cells. Overnight cultures were grown in 40 ml LB (100 μg ml^−1^ ampicillin [amp] and 1% [w/v] glucose) at 37°C. Two liter TB medium (100 μg ml^−1^ amp) was inoculated with the overnight culture and incubated at 37°C until an optical cell density (OD_600_) of 0.5–0.6 was reached. After cooling at 4°C for 20 min, cultures were induced with 0.25 mM IPTG, and grown for additional 16 hr at 20°C and 180 rpm. Cells were harvested by centrifugation, resuspended in lysis buffer and lysed by French press. After centrifugation (50,000 rcf for 30 min), the supernatant (supplemented with 2 mM MgCl_2_) was transferred to Ni‐NTA‐columns and washed with five CV wash buffer. The protein was eluted with elution buffer (wash buffer containing 300 mM imidazole) and concentrated. Pooled fractions, were separated on a Superdex 200 HiLoad 16/60 or 26/60 SEC column equilibrated with buffer (50 mM potassium phosphate, 200 mM potassium chloride, 10% (v/v) glycerol, 1 mM EDTA). All fractions containing monomeric ACP were pooled and concentrated to 10–20 mg ml^−1^.

#### 4.3.1 Protein concentration

Protein concentrations were calculated from the absorbance at 280 nm, which was recorded on a NanoDrop 2000c (Thermo Scientific). Extinction coefficients were calculated from the primary sequence without *N*‐formylmethionine with CLC Main workbench (Qiagen). Absorbance 1 g L^−1^ at 280 nm (10 mm): 1.053 for KS‐MAT and 0.475 for ACP.

### 4.4 Crystallization, data collection, and structure determination

Crystallization conditions for KS‐MAT were as previously published.^16^ Single‐crystals were obtained at 0.2 M potassium‐sodium tartrate, 25% (w/v) PEG 3350 at 20°C to sizes of about 75 × 75 × 75 μm^3^. Drops with the crystals were supplemented with 0.5 μl of 10 mM octanoyl‐CoA (Merck) for up to 2 min and subsequently treated with a cryosolution containing 20% (v/v) glycerol in the mother liquor. The crystal was then picked in a nylon fiber loop and vitrified into liquid nitrogen. Single wavelength X‐radiation diffraction dataset was collected at the Swiss Light Source (X06SA), and maintained at 100 K, while data were recorded onto a detector (DECTRIS EIGER 16M). Using the “http://goeiger.com” pipeline at X06SA, data reduction was performed within *XDS*,^59^ for indexing and integration, and *Aimless* 60 for scaling. The structural model of a monomer from the murine FAS KS‐MAT didomain complexed with Malonyl‐CoA (PDB accession code: http://firstglance.jmol.org/fg.htm?mol=5my0) was used to solve the phase problem using the program *Molrep*.^61^ After an initial rigid‐body refinement, the model was subjected to repeated cycles of restrained refinement with *REFMAC*5^62^ with manual model building using *Coot*.^63^ Data collection and refinement statistics are given in Table 1. Electron density maps were generated by Phenix.^64^

### 4.5 Thermal shift assay

Thermal shift assays were performed as previously reported.^17^ Briefly, 2 μl of protein solution (20 μM) were mixed with 21 μl of the respective buffer and 2 μl of SYPRO Orange protein gel stain (5,000× diluted), then fluorescence was measured from 5 to 95°C with a step of 0.5°C min^−1^, with excitation wavelength set to 450–490 nm, and emission wavelength to 560–580 nm. Data were analyzed with the software CFX Maestro 1.0.

### 4.6 α‐Ketoglutarate dehydrogenase coupled activity assay

The enzyme‐coupled assay was performed as previously published,^16^ which was adapted from Reference 50. Assays (octanoyl‐CoA transacylation of the KS^C161G^‐MAT^R606A^ variant and the series of response curves for the KS‐mediated transacylation of octanoyl moieties) were run in 96‐well f‐bottom microtiter plates (Greiner Bio‐one) and NADH fluorescence was monitored using a ClarioStar microplate reader equipped with a dispenser (BMG labtech) at the following settings; excitation: 348–20 nm; emission: 476–20 nm; gain: 1,900; focal height: 5.2 mm; flashes: 70; orbital averaging: 4 mm.

Within this study we reduced the assay volume first to 50 μl in 96‐well Half Area Microplates (Greiner Bio‐one) and then to 20 μl in 384‐well Small Volume HiBase Microplates (Greiner Bio‐one) for practical and financial reasons. The chain‐length specificity of the KS domain (Figure 5) and the series of response curves for the KS‐mediated transacylation of myristoyl moieties (Figure 6b) were measured in half‐area plates and 384‐well plates, respectively. New calibration curves with NADH and control measurements were performed for both smaller plate formats. Settings for the microplate reader were for half‐area plates: 348–20 nm; emission: 476–20 nm; gain: 1500; focal height: 5.6 mm; flashes: 17; orbital averaging: 1 mm; and for 384‐well plates: 348–20 nm; emission: 476–20 nm; gain: 1500; focal height: 11.9 mm; flashes: 17; orbital averaging: off.

Same procedures were used in preparation of assays even if they had different volumes. Briefly, four different solutions were prepared in assay buffer (50 mM sodium phosphate, 10% [v/v] glycerol, 1 mM DTT, 1 mM EDTA [pH 7.6], filtered and degased). Solution 1 (Sol 1) contained murine KS‐MAT in a 3.33‐fold or fourfold concentrated stock solution and supplemented with 0.1 mg ml^−1^ BSA. Solution 2 (Sol 2) contained 8 mM α‐ketoglutaric acid, 1.6 mM NAD^+^, 1.6 mM TPP, and 60 mU/100 μl αKGDH, representing a fourfold concentrated stock. Solution 3 (Sol 3) contained fourfold concentrated CoA‐esters, typically between 0.4–2,800 μM. Solution 4 (Sol 4) finally contained fivefold or fourfold concentrated murine ACP, typically between 20 and 800 μM. The components were pipetted in order: 30 μl Sol 1 (3.33‐fold), 25 μl Sol 2, and 25 μl Sol 3 for 96‐well plates; 15 μl Sol 1 (3.33‐fold), 12.5 μl Sol 2 and 12.5 μl Sol 3 for 96‐well half‐area plates and 5 μl Sol 1 (fourfold), 5 μl Sol 2 and 5 μl Sol 3 for 384‐well plates, followed by mixing. The transfer reaction was initiated by 20 and 10 μl Sol 4 (fivefold)) or 5 μl Sol 4 (fourfold), which was added by the dispenser. The final concentrations of all ingredients were 50 mM sodium phosphate, pH 7.6, 10% (v/v) glycerol, 1 mM DTT, 1 mM EDTA, 2 mM α‐ketoglutaric acid, 0.4 mM NAD^+^, 0.4 mM TPP, 15 mU/100 μl αKGDH, 0.03 mg ml^−1^ BSA, 100–200 nM KS‐MAT, 5–160 μM ACP, 0.1–700 μM X‐CoA (where X refers to the respective acyl‐moiety of the assay). The background noise of the assay setup was determined with assay buffer supplemented with 0.1 mg ml^−1^ BSA. Equidistant kinetic measurements were taken every 5–22 s for ca. 5 min at 30°C.

### 4.7 Transacylation kinetics of the MAT^R606A^ variant for octanoyl moieties

Determining the apparent Michaelis–Menten constant is an iterative process. Pre‐experiments were initially performed to approach the approximate value of *K*_m_. Final concentrations of enzyme and ACP were 100 nM and 60 μM, respectively. Eight data points were collected that cover substrate concentrations (Sol 3) of 0.2 × *K*_m_; 0.3 × *K*_m_; 0.5 × *K*_m_; 0.75 × *K*_m_; 1.25 × *K*_m_; 2 × *K*_m_; 3 × *K*_m_; 5 × *K*_m_. Every measurement was performed in technical triplicates and the corresponding background signal was subtracted. Experiments were setup in a way such that changes in signal remained linear during the time ranges of measurement. Data were collected in biological replicates (*n* = 4) and were fit with the Michaelis–Menten function using OriginPro 8.5 (OriginLab).

### 4.8 Chain‐length specificity of the KS domain

β‐Ketoacyl synthase‐mediated transacylation of acyl‐CoAs were measured at fixed enzyme (200 nM), ACP (75 μM), and acyl‐CoA (500 μM) concentrations. Turnover rates were determined by linear fit and error bars reflect the standard deviation from three biological replicates (*n* = 3).

### 4.9 Analysis of KS‐mediated transfer of octanoyl and myristoyl moieties

Initial velocities were determined for 12 different CoA‐ester concentrations at six (octanoyl‐CoA) and five (myristoyl‐CoA) fixed ACP concentrations. Enzyme concentration was 200 nM. Every measurement was performed in one biological replicate (*n* = 1) and the corresponding background signal was subtracted. Both series of response curves were globally fit using all data without any parameter constraints. The global fit was performed with OriginPro 8.5 (OriginLab) using the following equations for the ping–pong mechanism:

(1) $$v=\frac{k_{\mathrm{cat}}\left[{\mathrm{AT}}_{0}\right]\left[\text{XCoA}\right]\left[\mathrm{ACP}\right]}{\left[\text{XCoA}\right]K_{m}^{\mathrm{ACP}}+\left[\mathrm{ACP}\right]K_{m}^{\text{XCoA}}+\left[\text{XCoA}\right]\left[\mathrm{ACP}\right]}$$

Hill‐type variation:

(2) $$v=\frac{k_{\mathrm{cat}}\left[{\mathrm{AT}}_{0}\right]{\left[\text{XCoA}\right]}^{h}{\left[\mathrm{ACP}\right]}^{h}}{{\left[\text{XCoA}\right]}^{h}K_{\mathrm{ACP}}^{\prime }+{\left[\mathrm{ACP}\right]}^{h}K_{\text{XCoA}}^{\prime }+{\left[\text{XCoA}\right]}^{h}{\left[\mathrm{ACP}\right]}^{h}}$$

## CONFLICT OF INTEREST

The authors declare no competing interests.

## AUTHOR CONTRIBUTIONS

A.R. performed protein expression, purification experiments, enzymatic assays, and analyzed corresponding data. A.R. conceived the project. M.G. designed the research. A.H. performed kinetic experiments with the KS domain using octanoyl‐CoA under supervision of A.R. Crystallization was performed by A.R., A.H., and K.S.P. Crystal structure was solved by K.S.P.. A.R., K.S.P., and M.G. analyzed data and wrote the manuscript.

## Supporting information


**Appendix S1.** Supporting Information.

## Data Availability

PDB accession code for the atomic coordinates for the octanoyl‐bound murine KS‐MAT structure reported in this paper is http://firstglance.jmol.org/fg.htm?mol=6rop. X‐ray diffraction data are publicly available at https://dx.doi.org/10.5281/zenodo.2785017.
