# Supplementary material for: Type I fatty acid synthase trapped in the octanoyl‐bound state
Source: Protein Sci. 2020 Jan 11;29(2):589–605. doi: 10.1002/pro.3797 (PMC6954729; doi:10.1002/pro.3797)
Supplement: Supplementary file 1 — Appendix S1. Supporting Information. [file PRO-29-589-s001.pdf]

Supplementary Information

Type I fatty acid synthase (FAS) trapped in the octanoyl-bound state

Alexander Rittner, Karthik S. Paithankar, Aaron Himmler, and Martin Grininger*

Author affiliations

Institute of Organic Chemistry and Chemical Biology, Buchmann Institute for Molecular Life Sciences, Goethe University Frankfurt, Max-von-Laue-Str. 15, 60438 Frankfurt am Main, Germany

Correspondence

*grininger@chemie.uni-frankfurt.de

Table of contents:

Supplementary Figures:

Figure S1: Purification and crystallization of the KS-MAT didomain

Figure S2: Validation of ligand placement in the MAT domain

Figure S3: Stereo cartoon diagram to illustrate conformational changes in the MAT domain

Figure S4: Anisotropic movement of C-alpha atoms in the ferredoxin-like subdomain

Figure S5: Ramachandran plots for crucial residues in both subdomain linkers

Figure S6: Dynamics of the MAT domain is determined by flexible subdomain linkers

Figure S7: Rotational freedom of residue R606

Figure S8: Stability of select KS-MAT variants

Figure S9: Global Michaelis-Menten fit of KS-mediated transacylation data

Supplementary Tables

Table S1: Dimerization interface of the KS domain

Table S2: Primers and Plasmids

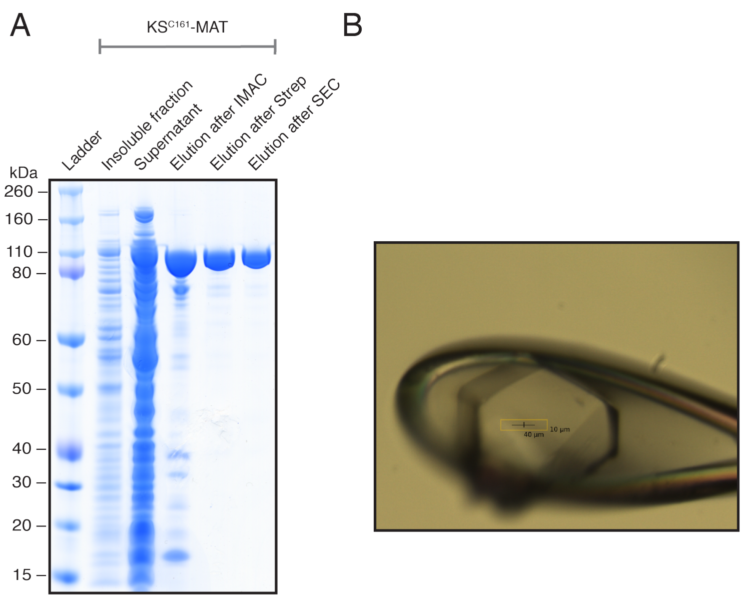

Figure S1: Purification and crystallization of the KS-MAT didomain. (A) SDS-PAGE (NuPAGE 4-12 % Bis-Tris) of the purification strategy of the KS-MAT didomain. A tandem purification using Ni-chelating and Strep-Tactin affinity chromatography was followed by size exclusion. (B) Photograph of the octanoyl-CoA soaked crystal within a nylon loop at the synchrotron.

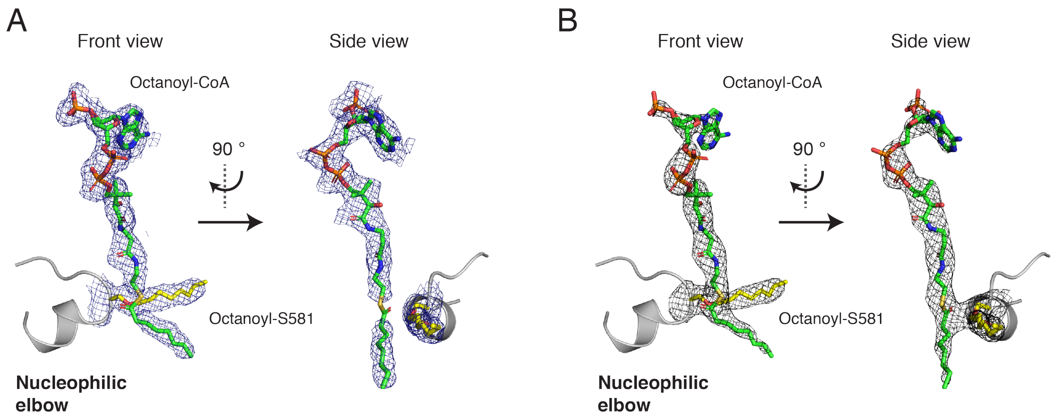

Figure S2: Validation of ligand placement in the MAT domain. Octanoyl-CoA and the covalent bound octanoyl-S581 were placed based on unbiased electron density maps. The FEM map (A) is shown in blue and the Polder map (B) in black. Contour levels are at 3 σ.

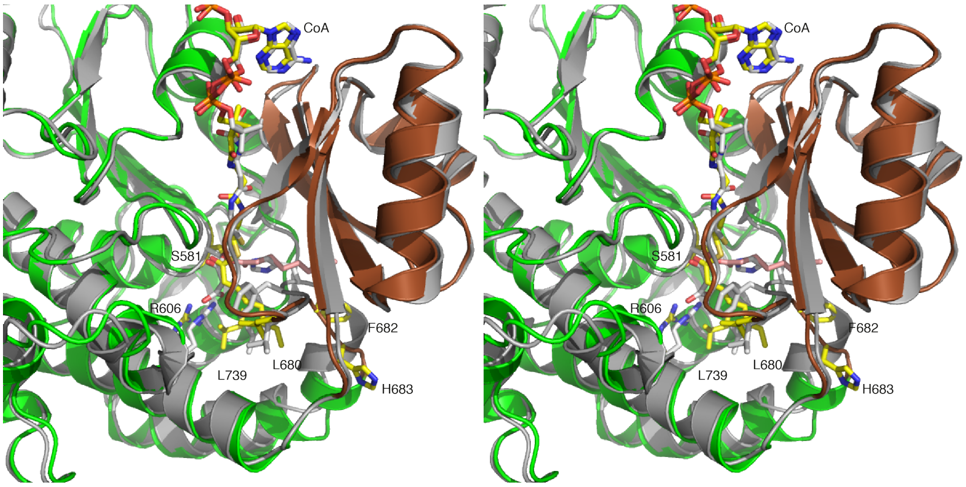

Figure S3: Stereo cartoon diagram to illustrate conformational changes in the MAT domain. Substrate bound chains (D) of the octanoyl-bound and malonyl-bound (grey) structures superimposed based on the α/β-hydrolase fold. Residues S581, R606, L680, F682, H683 and L739 and bound moieties are shown in stick representation. Green and brown colouring indicate the regions of α/β-hydrolase fold and the ferredoxin-like fold respectively for the octanoyl-bound model.

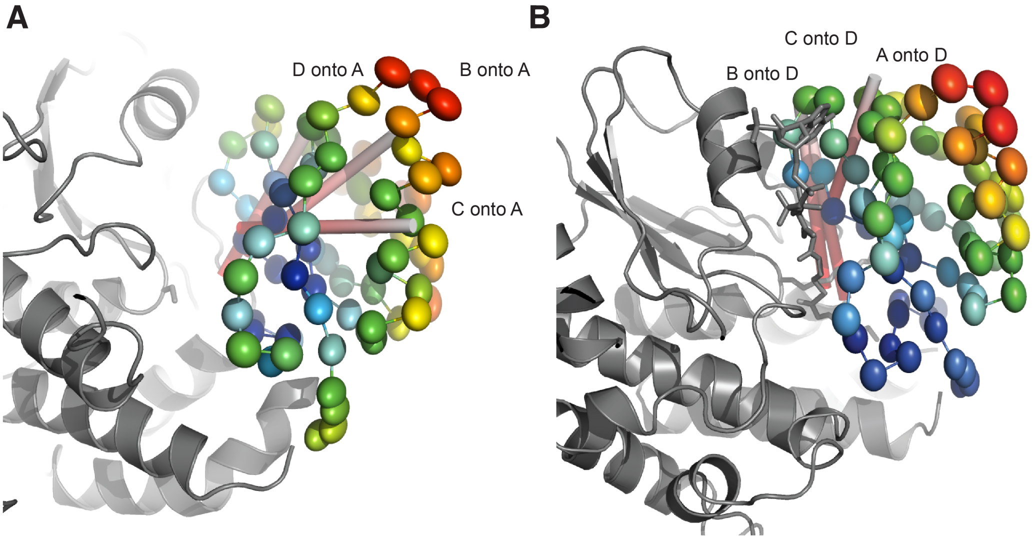

Figure S4: Anisotropic movement of C-alpha atoms in the ferredoxin-like subdomain. The anisotropic movement, as derived from the TLS tensors, is depicted by thermal ellipsoids colored by B-values (blue – low values; and red – high values) for the ferredoxin-like fold (616-684) region of chain A (A) and chain D (B). Rotation axes derived from superposition of ferredoxin-like fold (616-684) of chains B (A), C (B), and D (C) onto A (D) are shown in red-white cylindrical bars. For orientation, the rest of the KS-MAT chain is shown in grey.

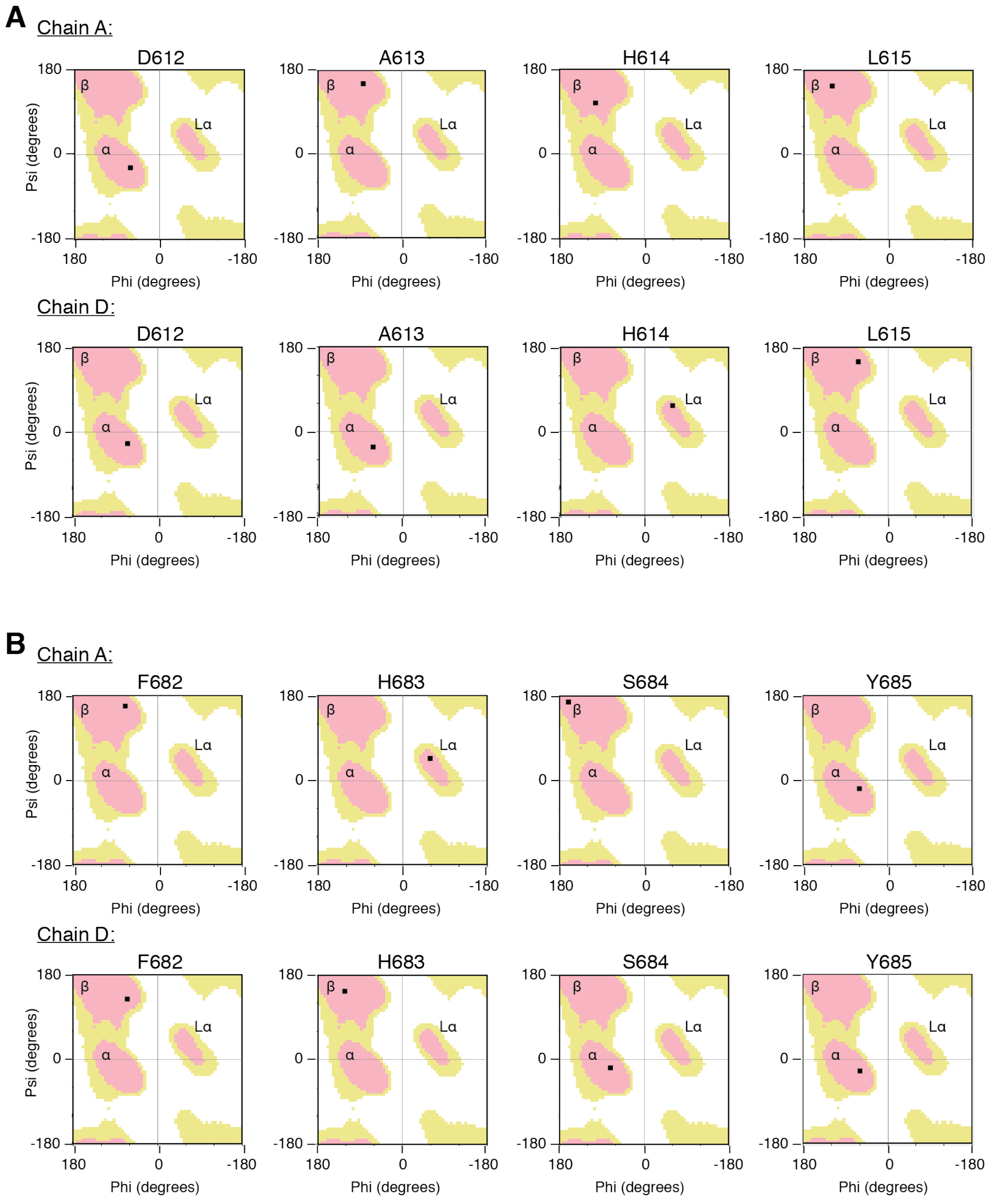

Figure S5: Ramachandran plots for crucial residues in both subdomain linkers. Significant changes in dihedral angles were seen for residues in linker 1 (A) 612-615 and linker 2 (B) 682-685 leading to changes to different allowed regions. Plots were created in coot. Used abbreviations indicate α – right-handed helical region, Lα – left-handed helical region and β – β-sheet region.

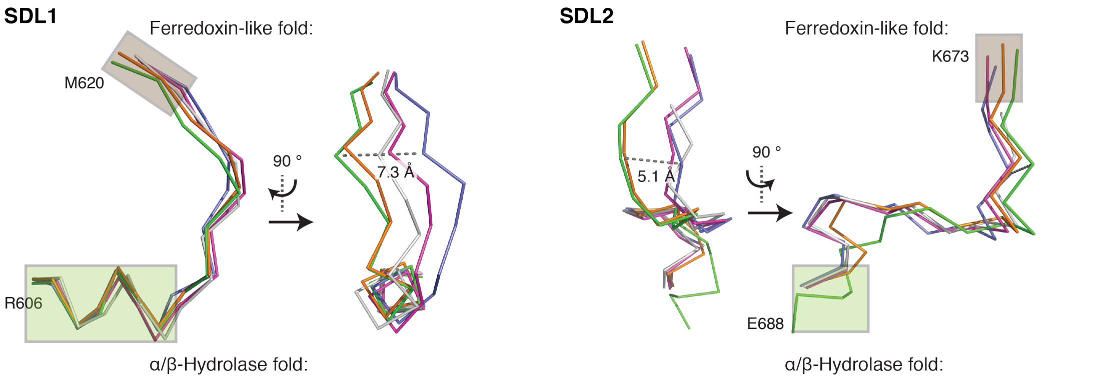

Figure S6: Dynamics of the MAT domain is determined by flexible subdomain linkers. SDL1 (612-617, left panel) and SDL2 (675-684, right panel) are shown as ribbons after α/β-hydrolase fold based alignments. Both linker stretches are shown from two different perspectives rotated by 90°. Chain A (blue), chain C (grey) and chain D (green) from the octanoyl-CoA soaked crystal (PDB code 6rop), malonyl-bound (orange) (PDB code 5my0; chain D) and apo human MAT (purple) (PDB code 3hhd; chain A) were used. Green and brown rectangles indicate secondary structure elements of the α/β-hydrolase- and ferredoxin-like subdomains, respectively.

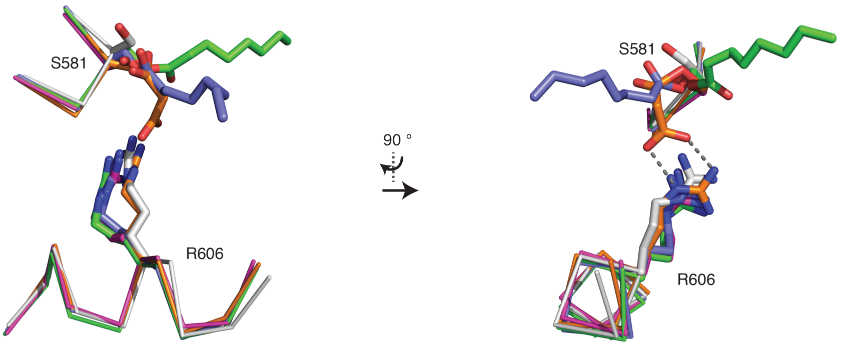

Figure S7: Rotational freedom of residue R606. α/β-Hydrolase based superposition (BB of 488-615) of chain A (blue), chain C (white) and chain D (green) from octanoyl-CoA soaked crystals (PDB code 6rop) with the malonyl-bound structure (orange) (PDB code 5my0; chain D) and of porcine FAS (purple) (PDB code 2vz9; chain A). Important residues S581 and R606 are shown in sticks with covalent modifications of the serine represented also in sticks. For clarity residue stretch 580-583 and 601-610 are depicted as ribbons. R606 in octanoyl-bound active sites adopts the same conformation as R606 in the porcine FAS, whereas R606 in chain C (unbound) possesses the rotameric state of the unbound active sites previously found in human KS-MAT (3hhd).[64]

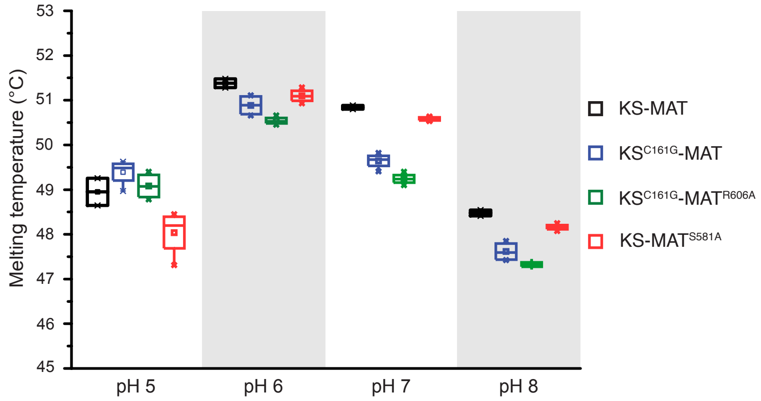

Figure S8: **Stability of select KS-MAT variants.** Melting temperatures were determined by a thermal shift assay as described in the Methods section. The four constructs were tested in phosphate buffers at different pH value. Four replicates are shown as dot plot.

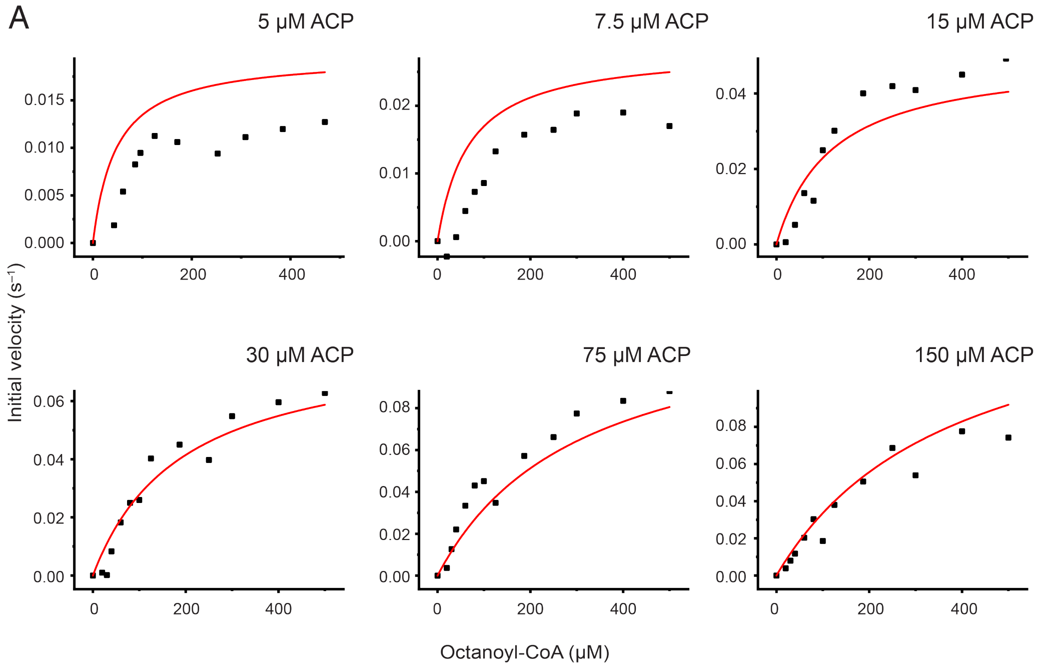

B

Substrate	$K_m^{X\text{-}CoA}$ (µM)	K_m^{ACP} (µM)	k_{cat} (s^{-1})
C8-CoA	511 ± 135	50 ± 12	0.22 ± 0.04
C14-CoA	231 ± 41	7 ± 2.5	0.07 ± 0.006

Figure S9: Global Michaelis-Menten fit of KS-mediated transacylation data. (A) Initial velocities were plotted against octanoyl-CoA (C8-CoA) concentrations at six fixed ACP concentrations. Data were fit globally with the Michaelis-Menten equation assuming a ping-pong bi-bi mechanism. (B) Absolute kinetic parameter derived from the respective global fits for C8- and C14-CoA, respectively. No parameters constraints were set for the fit function.

Table S1: Dimerization interface of the KS domain

Interface	Residues per side		Solvent-accessible interface area		Solvent energy gain per side	Hydrogen bonds	Salt bridges
	#	%	Å^2	%	kcal/mol		
KS chain A	73	8.6	2583	8.1	−14.9	38	0
KS chain B	71	8.5	2578	8.0	−14.8		0
KS chain C	72	8.5	2586	7.9	−14.5	34	0
KS chain D	75	8.8	2582	8.1	−15.0		0

Table S2: Primers and Plasmids

Number	Name	Length	Orientation	Sequence (5'3')	Construct Template
AR301	mMAT_S580X_rev	20	reverse	gtgcccaatgatgccgtcag	**pAR69/pAR70**
AR310	mMAT_S580A_for	38	forward	ggcatcattgggcacGccttgggagaggttgcctgtgg	**pAR69/pAR70**

Number	Construct	Name
pAR069*	KS-MAT	pAR69_STRI_m(KS_MAT)_H8_pET22b
pAR070*	KS^{C161G}-MAT	pAR70_StrepI_m(KS(C161G)_MAT)_H8_pET22b
pAR071*	KS^{C161G}-MAT^{R606A}	pAR71_StrepI_m(KS(C161G)_ATmut(R(606A))_H8_pET22b
pAR159	KS-MAT^{S581A}	pAR159_StrepI_m(KS_MAT(S581A))_H8_pET22b
pAR160	KS^{C161G}-MAT^{S581A}	pAR160_StrepI_m(KS(C161G)_MAT(S581A))_H8_pET22b
pAR352*	(holo)-ACP	pAR352_StrepII_mACP_H8_RBS_SFP_pET22b
